# Metabolic phenotypes of obese, overweight, and normal weight individuals and risk of chronic kidney disease: a systematic review and meta-analysis

**DOI:** 10.20945/2359-3997000000149

**Published:** 2019-07-11

**Authors:** Shahab Alizadeh, Hamed Esmaeili, Mohammad Alizadeh, Elnaz Daneshzad, Loghman Sharifi, Hossein Radfar, Mohammad Kazem Radaei

**Affiliations:** 1 Tehran University of Medical Sciences Department of Clinical Nutrition School of Nutritional Sciences and Dietetics Tehran University of Medical Sciences Tehran Iran Department of Clinical Nutrition, School of Nutritional Sciences and Dietetics, Tehran University of Medical Sciences (TUMS), Tehran, Iran; 2 Baqiyatallah Medical Sciences University Department of Nutrition and Food Hygiene Faculty of Health Baqiyatallah University of Medical Sciences Tehran Iran Department of Nutrition and Food Hygiene, Faculty of Health, Baqiyatallah University of Medical Sciences, Tehran, Iran; 3 Mazandaran University of Medical Sciences Department of Medical Surgical Nursing Nasibeh Nursing & Midwifery School Mazandaran University of Medical Sciences Sari Iran Department of Medical Surgical Nursing, Nasibeh Nursing & Midwifery School, Mazandaran University of Medical Sciences, Sari, Iran; 4 Tehran University of Medical Sciences Department of Community Nutrition School of Nutritional Sciences and Dietetics Tehran University of Medical Sciences Tehran Iran Department of Community Nutrition, School of Nutritional Sciences and Dietetics, Tehran University of Medical Sciences (TUMS), Tehran, Iran; 5 Tehran University of Medical Sciences Department of Cellular and Molecular Nutrition School of Nutritional Sciences and Dietetics Tehran University of Medical Sciences Tehran Iran Department of Cellular and Molecular Nutrition, School of Nutritional Sciences and Dietetics, Tehran University of Medical Sciences (TUMS), Tehran, Iran; 6 Kharazmi University Department of Sports Biomechanics hysical Education and Sport Science College Kharazmi University Tehran Iran Department of Sports Biomechanics, hysical Education and Sport Science College, Kharazmi University, Tehran, Iran

**Keywords:** Metabolic health, obesity, chronic kidney disease, meta-analysis

## Abstract

**Objective:**

Chronic kidney disease (CKD) risk is inconsistent in the normal-weight, overweight, and obese individuals due to the heterogeneity of metabolic status. This meta-analysis aimed to examine the combined effects of body mass index (BMI) and metabolic status on CKD risk.

**Materials and methods:**

The MEDLINE, EMBASE, and Web of Knowledge databases were systematically searched up to March 2019 to identify all eligible studies investigating the CKD risk (defined as GFR < 60 mL/min per 1.73 m^2^ and/or microalbuminuria or proteinuria) associated with the body size phenotypes which are known as metabolically unhealthy normal-weight (MUNW), metabolically healthy overweight (MHOW), metabolically unhealthy overweight, metabolically healthy obese (MHO) and metabolically unhealthy obese (MUHO). The classification of subjects in included studies as metabolically unhealthy was based on the presence of three components of metabolic syndrome. BMI categorization was based on the criteria of included studies. The risk estimates and 95% confidence intervals (CIs) were extracted and pooled using random effects analysis.

**Results:**

A total of 9 prospective cohort studies with 128773 participants and 4797 incident cases were included in the meta-analysis. Compared with healthy normal-weight individuals as reference, MUNW and MHO subjects showed an increased risk for CKD events with a pooled RR of 1.58 (95% CI = 1.28-1.96) in MUNW and 1.55 (95% CI = 1.34-1.79) in MHO persons. Also, MHOW was at increased risk for CKD (RR = 1.34, 95% CI = 1.20-1.51). MUHO individuals were at the highest risk for the development of CKD (RR = 2.13, 95% CI = 1.66-2.72).

**Conclusions:**

Individuals with metabolic abnormality, although at normal-weight, have an increased risk for CKD. Healthy overweight and obese individuals had higher risk; refuting the notion that metabolically healthy overweight and obese phenotypes are benign conditions.

## INTRODUCTION

Chronic kidney disease (CKD), which nearly doubled as a cause of death around the world between 1990 and 2010 and was the 18^th^ highest cause of death worldwide in 2010 (1), is a worldwide health problem with increasing incidence and prevalence, high costs and poor outcomes (2). Besides being a major risk factor for end-stage renal disease (ESRD), CKD is an important risk factor for cardiovascular disease (3) and increased mortality rates (4). Therefore, prevention and management of CKD by identifying and treating its risk factors are of critical urgency.

One of the major risk factors for CKD is obesity (5) and obesity-related metabolic disorders such as diabetes, hypertension, and metabolic syndrome (6,7). Accumulating evidence has suggested that, initially, obesity causes renal vasodilation and glomerular hyperfiltration, which acts as a compensatory mechanism to maintain sodium balance despite increased tubular reabsorption. Subsequently, these compensations, along with increased arterial pressure and metabolic abnormalities, may eventually lead to glomerular injury and initiate a slowly developing vicious cycle that exacerbates hypertension and worsens renal injury (8). Nevertheless, the role of obesity in kidney insufficiency is controversial (9) and depend, in part, on the clustering of metabolic and cardiovascular risk factors (10). The glomerular filtration rate is dependent on the duration of the obesity, where a compensatory hyperfiltration occurs in the initial years to meet the heightened metabolic demands, and a decline in the glomerular filtration rate may occur in long-term, due to the increase in intraglomerular pressure (8). Most studies (11-15), but not all (16,17), have shown that obese subjects exhibit lower glomerular filtration rate (GFR). These discrepancies might be due to the heterogeneity of obesity phenotypes (18). During 1980 to 2000, epidemiological studies demonstrated that not all obese subjects display a clustering of metabolic and cardiovascular risk factors, and, likewise, not all lean subjects present a healthy metabolic and disease-free profile (19,20). Accordingly, recently attention was drawn to this concept and different body size phenotypes were defined (21) based on metabolic health status (22). Metabolically healthy obesity (MHO) is one of the most intriguing phenotypes in this regard, which its prevalence depending on the definitions used for obesity and metabolic health, varies from 6.0% to 38.4% in different populations[23]. Individuals with MHO display a favorable metabolic profile that is characterized by a high level of insulin sensitivity, favorable lipid profiles, a low incidence of hypertension, satisfactory fat distribution, and a low level of systemic inflammatory responses (23,24). Another body size phenotype, which is known as metabolically unhealthy and normal-weight (MUNW), include normal-weight individuals who based on standard weight tables are not obese (BMI < 25 kg/m^2^), but express metabolic abnormalities like an increased levels of adiposity and insulin resistance and a higher susceptibility to type 2 diabetes and cardiovascular diseases (CVD) (25,26). Moreover, elderly people with MUNW phenotype exhibited a higher risk of all-cause and CVD mortality (26).

Whether different phenotypes of obesity have different effects on the risk of CKD is still debatable. To date, observational studies have shown inconsistent effects of obesity phenotypes on the risk of developing CKD. A single study might have low statistical power due to small sample size and other limitations. Thus, the current meta-analysis was conducted to assess the relation of different phenotypes of body size to CKD risk.

## MATERIALS AND METHODS

Our meta-analysis was conducted according to the Meta-analysis of Observational Studies in Epidemiology (MOOSE) guidelines (27).

### Search strategy

We searched for all published observational studies that described the associations of different phenotypes of body size with the risk of CKD incident. A systematic literature search was performed using the MEDLINE, EMBASE, and Web Of Knowledge databases and was supplemented with the manual review of the reference list of obtained articles up to March, 2019. The following terms were used: ((((((((“Obesity”[Mesh] (28) OR “Body Mass Index”[Mesh]) OR body mass index[Title/Abstract]) OR obesity[Title/Abstract]) OR obese[Title/Abstract]) OR overweight[Title/Abstract]) OR normal weight[Title/Abstract]) AND (metabolic[All Fields] OR metabolically[Title/Abstract])) OR ((healthy[Title/Abstract] OR unhealthy[Title/Abstract]) OR benign[Title/Abstract])) AND ((((((“Kidney Failure, Chronic”[Mesh] OR “Renal Insufficiency, Chronic”[Mesh]) OR chronic kidney disease[Title/Abstract]) OR CKD[Title/Abstract]) OR chronic renal disease[Title/Abstract]) OR chronic kidney insufficiency[Title/Abstract]) OR chronic renal failure[Title/Abstract]). To find studies investigating the combined effect of BMI and metabolic status on the risk of CKD and remove irrelevant studies, a specific search strategy was applied. No language restriction was applied for searching and study inclusion.

### Eligible criteria

Studies were considered eligible for meta-analysis if they met the following criteria: the study had a prospective cohort design; the exposures were the metabolically healthy and metabolically unhealthy phenotypes of body size and outcomes was incident of CKD; stratified subjects according to BMI categories; had one reference group in the normal-weight healthy range; reported hazard ratios (HRs), relative risks (RRs) or odds ratios (ORs) for incident of CKD; CKD incident diagnosis; and reported the criteria used for defining subject as metabolically healthy or metabolically unhealthy. Literature reviews, cross-sectional studies, case reports, republished data, and animal studies were excluded. The application of these criteria yielded 6 studies eligible for the meta-analysis.

### Data extraction

The following information was collected from each study: the author’s name, publication year, country of origin, study design, ethnicity, gender, mean or range of age, sample size, duration of the follow-up, numbers of CKD cases, definition of metabolically healthy and metabolically unhealthy phenotypes, confounding factors that were adjusted for in the multivariable analysis, and hazard ratios (HRs), relative risks (RRs) or odds ratios (ORs) with 95% confidence intervals (CIs). Data were extracted independently by two investigators, and the disagreements during the data extraction were resolved by discussion among all reviewers. CKD diagnosis as outcome was based on GFR < 60 mL/min per 1.73 m^2^ and/or microalbuminuria or proteinuria during at least 3 months of follow-up. The classification of subjects in included studies as metabolically unhealthy was based on the presence of at least three components of metabolic syndrome. BMI categorization was based on the criteria of included studies

### Quality assessment

The quality of the studies was independently assessed by the same two reviewers and any disagreement was resolved by discussion between the two investigators. The Newcastle–Ottawa Scale (NOS) was applied to assess the quality of included studies (29). This quality assessment tool judges studies on the basis of a star system, ranging from 0 to 9 stars and includes the areas of selection, comparability, and outcome. Reports scoring 6 to 9 were classified as high quality.

### Categorization of different phenotypes of obesity

The classification of subjects in included studies as metabolically unhealthy was based on the presence of at least 3 components of metabolic syndrome by criteria from the National Cholesterol Education Program’s Adult Treatment Panel III (fasting triglyceride level ≥ 1.69 mmol/L (150 mg/dL); HDL-C < 1.04 mmol/L (40 mg/dL) in men or < 1.29 mmol/L (50 mg/dL) in women or lipid-lowering medication use; fasting glucose level ≥ 5.6 mmol/L (100 mg/dL) or antidiabetic medication use; systolic blood pressure 130 mmHg, diastolic blood pressure 85 mmHg, or use of antihypertensive medication; waist circumference > 88 cm for women and > 102 cm for men) (30) or International Diabetes Federation (HDL cholesterol level < 1.04 mmol/L [< 40 mg/dL] in men or < 1.29 mmol/L [< 50 mg/dL] in women; fasting triglyceride level < 1.69 mmol/L [≥ 150 mg/dL]; fasting glucose level ≥ 5.6 mmol/L [≥ 100 mg/dL] or glucose lowering medication; systolic blood pressure ≥ 130 mmHg, diastolic blood pressure ≥ 85 mmHg, antihypertensive medication, or history of hypertension; and waist circumference ≥ 94 cm in men or ≥ 80 cm in women) (31). The subjects of the included original studies were stratified into normal-weight, overweight, and obese categories and with or without metabolic syndrome to describe different phenotypes of body size as the following: metabolically healthy with normal-weight (MHNW), metabolically unhealthy with normal-weight (MUNW), metabolically healthy with overweight (MHOW), metabolically unhealthy with overweight (MUHOW), metabolically healthy with obesity (MHO), and metabolically unhealthy with obesity (MUHO).

### Statistical analysis

Pooled relative risk with 95% CI was calculated for CKD events as outcomes for the MUNW, MHOW, MUHOW, MHO, and MUHO phenotypes using the number of events in individuals with MHNW phenotype as the control group. RRs were combined based on Mantel-Haenszel method. Heterogeneity among the studies was evaluated by the I^2^ statistics [I^2^ = (Q-df)/Q × 100%; I^2^ < 25%, no heterogeneity; I^2^ = 25-50%, moderate heterogeneity; I^2^= 50-75%, large heterogeneity, I^2^ > 75%, extreme heterogeneity] (32). The heterogeneity was considered significant if I^2^> 50% (p < 0.1). All analyses were performed using the random-effects model (33,34). Visual inspection of asymmetry in funnel plots, and Egger’s (35) and Begg’s test (36) were used to evaluate small-study bias (p < 0.05 was considered as statistical significance). All statistical tests for current the meta-analysis were performed with STATA (version 14.0; Stata Corporation, College Station, TX).

## RESULTS

### Study characteristics

**Figure 1:**
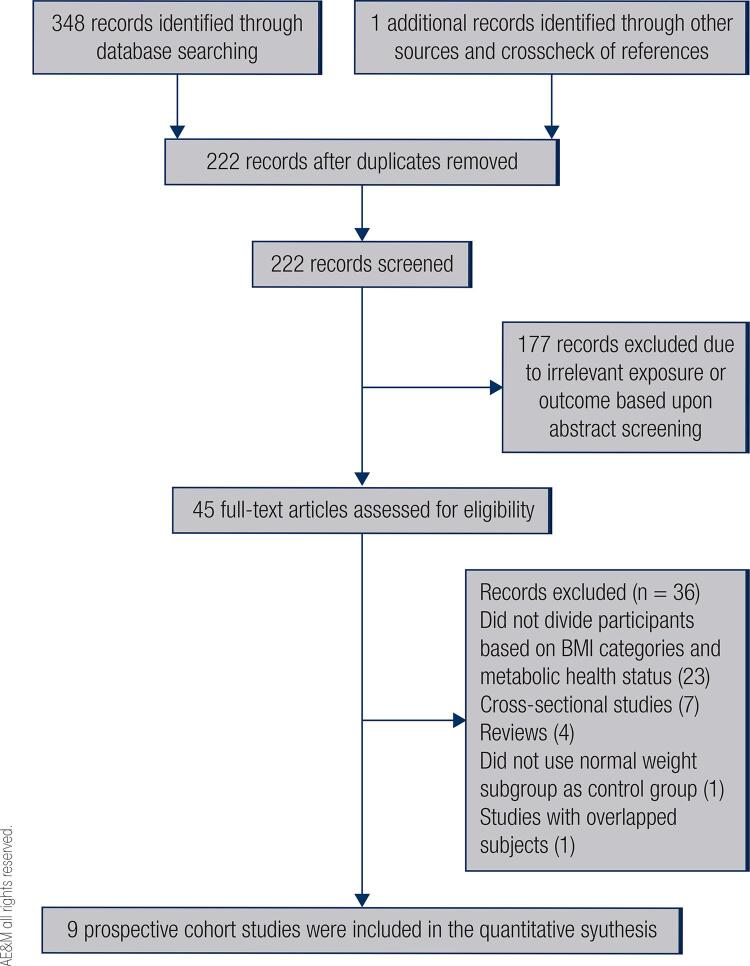
Outline of the systematic study selection process for the meta-analysis.

A total of 349 studies were identified by the literature search which one of them was identified through crosscheck of references (37). The flow diagram describing the process of screening and excluding studies with specific reasons is presented in Figure 1. Of the excluded studies, 127 were duplicate publications; 177 had irrelevant exposure or outcome; 23 did not divide subjects according to BMI categories and metabolic health status; 4 were review studies; 1 did not use MHNW participants as control group; 1 was a study with overlapped subjects, and 7 were cross-sectional reports. The primary eligibility process yielded 8 studies (28,38-44) and crosscheck of the references of reviews and included studies, and other databases search yielded 1 additional study (37). Finally, a total of 9 studies, comprising 128773 participants and 4797 incident cases, were included in the quantitative meta-analysis based on the inclusion criteria for CKD risk related to the different phenotypes of body size. Of these, four were from South Korea (28,37,38,42), one was from Iran (39), two were from China (40,43), one in USA (44) and one was from Japan (41). The sample size of the included studies varied from 1881 to 62249 participants and the follow-up duration ranged from 3.2 to 14 years. Three studies (37,39,42) had estimated GFR by the Modification of Diet in Renal Disease (MDRD) Study equation, one study by the Chronic Kidney Disease Epidemiology Collaboration (CKD-EPI) equation (38), one study by the Japanese Society of Nephrology equation (41), and one study by a modified Chinese equation (40). In the assessment of metabolic abnormality, the classification of subjects in these studies as metabolically unhealthy was based on the presence of metabolic syndrome (28,38,40-44), and by the presence of metabolic syndrome combined with insulin resistance (37,39). The definition of metabolic syndrome was also based on criteria by the National Cholesterol Education Program’s Adult Treatment Panel (NCEP ATPIII) and International Diabetes Federation (IDF) criteria. The results of the included studies were adjusted for the most potential confounders, including age, sex, physical inactivity, and smoking status. Furthermore, the reference category was a MHNW group in all studies; however, specific cut-offs were varied. In accordance with the NOS quality assessment scale, all studies achieved at least 7 stars, showing overall good quality. Table 1 shows detailed information about the studies included in the meta-analysis.

**Table 1:** Characteristics of studies included in the meta-analysis

Study	Country	Duration of follow-up	Mean age (% male)	Sample size and incident cases (Ntotal/NCKD)	Metabolic health criteria	Definition of BMI categories (kg/m^2^)	CKD criteria (Equation)	Adjusted variables in analyses	Quality (/9)
Jung and cols., 2015 (38)	South Korea	3.2 years	47.9 (60% male)	Total (41194/356) MHNW (20329/86) MUNW (4835/56) MHO (8587/89) MUHO (7443/ 125)	Metabolic syndrome: ≤ 1 of the ATP III criteria: (1) a systolic BP ≥ 130 mmHg and/or a diastolic BP ≥ 85 mmHg, or on antihypertensive treatment; (2) TG ≥ 1.7 mmol/l; (3) FPG ≥ 5.6 mmol/l or on antidiabetic treatment; and (4) HDL-C < 1.0 mmol/l in men and < 1.3 mmol/l in women.	Normal-weight < 25 Obese ≥ 25	GFR < 60 (CKD-EPI)	Age, sex, baseline GFR, history of cardiovascular disease, drinking, smoking, and exercise habits, alanine aminotransferase (ALT), γ-glutamyltransferase (GGT), LDL-C, uric acid, and hs-CRP	9
Mottaghi and cols., 2015 (39)	Iran	9.4 years	40.3 (45.2% male)	Total (5672/1162) MHNW (1817/246) MUNW (202/70) MHOW (1603/314) MUHOW (786/ 212) MHO (500/116) MUHO (764/204)	1) Metabolic syndrome: ≤ 2 of the ATP III criteria: (1) FBG ≥ 100 mg/dl or medication use for treatment of impaired fasting glucose; (2) TG ≥ 150 mg/dl or medication use; (3) HDL-C < 40 mg/dl for men and <50 for women or medication use; (4) BP ≥ 135/85 mmHg or medication use and (5) WC ≥ 95 cm for both sexes. 2) having diabetes	Normal-weight < 25 Overweight 25-29.9 Obese ≥ 30	GFR < 60 (MDRD)	Age, sex, smoking, hypertension , waist circumference	9
Cao and cols., 2015 (40)	China	5 years	46.5 (36.7% male)	Total (6852/740) MHNW (3632/248) MUNW (232/16) MHOW (1852/248) MUHOW (656/ 104) MHO (204/56) MUHO(276/68)	Metabolic syndrome: ≤ 2 of the ATP III criteria: 1) WC: ≥ 90 cm (males) or ≥ 80 cm (females); 2) elevated TG: ≥ 1.69 mmol/l or the use of lipid medications; 3) elevated BP: systolic BP ≥ 130 mmHg, or diastolic BP ≥ 85 mmHg, or the use of antihypertensive medications; 4) elevated FPG: ≥ 5.6 mmol/l or the use of diabetes medications; 5) reduced HDL-c: < 1.04 mmol/l (male) or < 1.29 mmol/l (female)	Normal-weight < 24 Overweight 24-27.9 Obese ≥ 28	GFR < 60 (modified Chinese equation) or proteinuria > 30 mg/dL	age, sex, smoking, plasma low-density lipoprotein cholesterol level, medication use, and physical inactivity	8
Hashimoto and cols., 2015 (41)	Japan	8 years	46.58 (58% male)	Total (3136/123) MHNW (2122/56) MUNW (445/30) MHO (302/8) MUHO (267/ 29)	Metabolic syndrome: ≤ 1 of the International Diabetes Federation. criteria: 1) impaired fasting glucose or diabetes (FPG ≥ 100 mg/dl or who were under medical treatment), 2) hypertension (systolic BP ≥ 130 mmHg and/or a diastolic BP ≥ 85 mmHg or who were under medical treatment), 3) hypertriglyceridemia (TG ≥ 150 mg/dl or treatment of hyperlipidemia), and 4) low HDL cholesterol concentration HDL < 40 mg/dl in men and < 50 mg/dl in women)	Normal-weight < 25 Obese ≥ 25	GFR < 60 (Japanese Society of Nephrology equation)	Age, sex, smoking status and alcohol use, creatinine, uric acid, systolic blood pressure, HDL cholesterol, and impaired fasting glucose or diabetes.	9
Song and cols., 2015 (42)	South Korea	3.7 years	44.1 (37% male)	Total (1881/62)	metabolic syndrome: ≤ 2 of the following criteria: WC ≥ 90 cm for men and ≥ 85 cm for women; BP ≥ 130/85 mmHg or history of hypertension; FPG ≥ 5.6 mmol/L (100 mg/dL); HDL < 1.03 mmol/L (40 mg/dL) for men or 1.29 mmol/L (50 mg/dL) for women; and TG ≥ 1.7 mmol/L (150 mg/dL)	Normal-weight < 25 Obese ≥ 25	GFR < 60 (MDRD)	sex, alcohol use, smoking amount, and physical activity at baseline	7
Chang and cols., 2016 (37)	South Korea	5 years	36.1 (50.5% male)	Total (62249/906) MHNW (36490/456) MHOW (13149/232) MHO (8149/177)	Metabolic syndrome and insulin sensitivity: Metabolic health was defined as a HOMA-IR < 2.5 and the absence of any component of the metabolic syndrome.	Normal-weight < 23 Overweight 23-24.9 Obese ≥ 25	GFR < 60 (MDRD)	age, sex, study center, year of screening examination, smoking status, alcohol intake, and physical activity at baseline	7
Lin and cols., 2017 (43)	China	3.9 years	59.5 (38.8% male	Total (2491/243) MHNW (758/34) MUNW (485/57) MHO (441/37) MUHO (807/ 115)	Metabolic syndrome: ≤ 2 of the ATP III criteria	Normal-weight < 25 Obese ≥ 25	GFR < 60 Or urinary albumin-to creatinine ratio ≥ 30 mg/g	age and sex, baseline estimated glomerular filtration rate, history of cardiovascular disease, drinking, smoking and exercise habits, education levels, and occupation, alanine aminotransferase, cglutamyltransferase, uric acid, and homeostasis model assessment of insulin resistance, high-sensitivity C-reactive protein.	8
Nam and cols., 2018 (28)	Korea	9.3 years	51.9 (47.9% male	Total (3249/782) MHNW (1568/77) MUNW (478/85) MHO (384/39) MUHO (819/ 156)	Metabolic syndrome: ≤ 2 of the ATP III criteria	Normal-weight < 25 Obese ≥ 25	GFR < 60 mL	age, sex, residence, history of cardiovascular disease, smoking status, alcohol intake, and new development of malignancy and cardiovascular disease during follow-up period, ALT, GGT, HOMA-IR, albumin, CRP, and proteinuria	9
Echouffo-Tcheugui and cols., 2019 (44)	USA	14 years	44.7 (47.9% male	Total (2049/423) MHNW (848/129) MUNW (162/22) MHO (608/145) MUHO (431/ 127)	Metabolic syndrome: ≤ 2 of the ATP III criteria	Non-obese < 30 Obese ≥ 30	GFR < 60 mL and/or microalbuminuria	age, sex, current smoking	9

MHNW: metabolically healthy normal weight; MUNW: metabolically unhealthy normal weight; MHOW: metabolically healthy overweight; MUHOW: metabolically unhealthy overweight, MHO: metabolically healthy obese; MUHO: metabolically unhealthy obese GFR: glomerular filtration rate; HOMA-IR: homeostatic model assessment of insulin resistance; LDL-C: low-density lipoprotein cholesterol; hs-CRP: high-sensitivity C-reactive protein; WC: waist circumference; ALT: alanine aminotransferase; GGT: gamma-glutamyltransferase; TG: triglyceride; HDL: high-density lipoprotein; FPG: fasting blood glucose; BP: blood pressure; ATP III: Adult Treatment Panel III; CKD-EPI: Chronic Kidney Disease Epidemiology Collaboration equation; MDRD: Modification of Diet in Renal Disease Study equation.

### Metabolically unhealthy normal-weight (MUNW) phenotype and CKD risk

There were 8 prospective studies (38-44), with a total of 2607 cases and 59860 participants concerning MUNW phenotype and CKD risk. The result of the pooled RR is presented in **Supplemental file 1** and Table 2. When all eligible studies were pooled, the analysis revealed that the MUNW phenotype was associated with a 58% increased risk of CKD, compared with the MHNW phenotype (RR = 1.58, 95%CI = 1.28-1.96), and a significant variability was observed (I2 = 45.4%, P = 0.077). There was no evidence for small-study bias on the basis of the Egger’s regression test (t = -0.01, p = 0.994) (Table 2).

**Table 2:** Meta-analysis for the risk of chronic kidney disease among different phenotypes of body size compared to metabolically healthy normal weight phenotype Metabolically healthy overweight (RR = 1.34, 95% CI = 1.20-1.51) was at increased risk for CKD.

Subgroup	NO of studies					publication bias	

		Test of association		Test of heterogeneity		Egger’s	

		OR	95%C	I^2^ (%)	P	t	P
MUNW	8	1.58	1.28-1.96	45.4	0.07	-0.01	0.994
MHOW	3	1.34	1.20-1.51	0.0	0.70	-0.38	0.767
MUHOW	2	1.53	0.95-2.48	69.5	0.07	-0.28-	0.652
MHO	9	1.55	1.34-1.79	32.1	0.16	0.14-	0.896
MUHO	8	MUHO	1.66-2.72	68.4	0.002	1.38	0.216

MUNW: metabolically unhealthy normal weight; MHOW: metabolically healthy overweight; MUHOW: metabolically unhealthy overweight, MHO: metabolically healthy obese; MUHO: metabolically unhealthy obese.

### Metabolically healthy overweight and metabolically unhealthy overweight phenotypes and CKD risk

There were 3 prospective cohort studies (37,39,40), comprising 74773 participants and 2808 incident cases regarding the association between MHOW phenotype and CKD risk. Moreover, there were 2 studies (39,40) with 1902 cases and 12524 participants addressing the risk associated with MUHOW phenotype. Individuals with MHOW phenotype had a significantly increased the risk for CKD events compared with MHNW persons (RR = 1.34, 95% CI = 1.20-1.51) (Table 2 and **Supplemental file 2**). All but 1 study reported a significant difference between the groups. There was no between-study heterogeneity (I^2^ = 0.0%, P = 0.705), with no evidence of small-study bias on the Egger’s regression test (t = -0.38, p = 0.767). Furthermore, compared to the MHNW phenotype, MUHOW individuals showed no significant association with risk of CKD (RR = 1.53, 95% CI = 0.95-2.48) (**Supplemental file 3**), with no evidence of significant heterogeneity (I2 = 69.5%, P = 0.070) (Table 2).

### Metabolically healthy obese and metabolically unhealthy obese phenotypes and CKD risk

A total of 9 prospective studies (28,37-44), involving 122417 participants and 3570 cases, were included in the analysis of CKD risk in relation to MHO phenotype. In addition, there were 8 studies (28,38-44) with 2841 cases and 60792 participants regarding the association between MUHO phenotype and risk of incident CKD. The results showed that the MHO phenotype was associated with a 55% increased risk of CKD, compared to the MHNW phenotype (RR = 1.55, 95% CI = 1.34-1.79) (**Supplemental file 4**). There was no significant evidence for heterogeneity in the effect sizes for these associations across studies (I2 = 32.1%, P = 0.161), and no evidence of small-study bias was detected (t = -0.14, p = 0.896). In comparison, the corresponding pooled RR in the MUHO individuals was 2.13 (95% CI = 1.66-2.72) (Table 2 and **Supplemental file 5**). However, there was significant heterogeneity in the individual estimates when the magnitude of the association was evaluated (I2 = 68.4%, P = 0.002), with no evidence of small-study bias according to the Egger’s regression test (t = 1.38, p = 0.216) (Table 2).

## DISCUSSION

In the present meta-analysis of 9 prospective cohort studies with 128773 participants, we addressed the combined effect of BMI and metabolic health status on the risk of CKD. The results revealed that compared with MHNW subjects, the overweight and obese individuals even in the absence of overt metabolic abnormalities, had significantly increased the risk for CKD.

The differentiation of metabolically healthy overweight/obesity and metabolically impaired overweight/obesity has been suggested to have important implications for therapeutic medical decision-making (45,46). As the main finding, this meta-analysis showed that overweight and obese individuals are at higher risk for CKD regardless of their metabolic status, refuting the notion that overweight and obesity without metabolic abnormalities are benign conditions. There are several common biological states directly linking obesity to kidney dysfunction independent of metabolic risk factors, including hemodynamic changes, oxidative stress, hormonal effects, and activation of the renin-angiotensin-aldosterone system, which are common in overweight and obesity states and could changes renal hemodynamic via activation of the renal sympathetic system (37,47-49). In addition, adipose tissue functions as an active endocrine organ and dysregulation in the production of adipose tissue-derived adipokines and cytokines such as leptin, adiponectin, tumor necrosis factor-α, interleukin-6, and plasminogen activator inhibitor-1 may also be involved in the pathogenesis of CKD among overweight and obese persons (48,50-52). The mechanisms determining metabolic status in individuals at the same BMI are not well known. The main proposed factor is the pattern of fat distribution, with excess visceral fat being more detrimental for metabolically unhealthy status than excess subcutaneous fat (54). In addition, emerging evidence has shown that ethnicity, genetic and epigenetic programming (54-56), and behavioral and environmental factors (53,57) may also be involved. Despite normal BMI, individuals with MUNW phenotype have metabolic disturbances, which are characterized by having a high body fat percentage, especially visceral fat, and a low level of physical activity, a low lean body mass, a low resting metabolic rate, and low insulin sensitivity (58). Previous studies have suggested that East-Asians have a higher risk of MUNW than Caucasians, and this ethnic difference may be attributable to higher abdominal fat accumulation in Asians than Caucasians in the same BMI range (50). Given the association between the MUNW phenotype and increased risk of CKD and other diseases (59-62), early identification of MUNW individuals, who often elude screening as they are not perceived as high risk, is important to predict and prevent renal insufficiency, particularly in East-Asian population.

The findings of this meta-analysis will help establish whether MUNW and seemingly healthy overweight and obese populations face an increased risk for CKD. The main strength of the current study is its relatively large pooled sample size, enabling the determination of robust estimates for the relationship between the 6 BMI– metabolic categories and related disease that could not be estimated precisely in individual studies. Moreover, the included studies were all prospective cohort in design, which minimized the possibility of selection bias and recall bias. However, some limitations of our study should be discussed. First, because of the limited available published literature, the number of studies included in this meta-analysis is relatively small. Second, the duration of exposure to the current metabolic phenotype and longitudinal changes in metabolic status and BMI were not reported in the studies, which could partially affect the estimated risk. Studies have shown that metabolic health might be a transient condition among obese people (62) and appears to be accumulating (63-65). It cannot be ignored that some healthy persons at baseline may develop metabolic risk factors and overt disease over time. Thus, it is likely that metabolic and weight changes, rather than cross-sectional weight status, impact CKD risk most strongly. Third, data regarding relative change in GFR and proteinuria during the follow-up periods were not reported in the included studies. Fourth, significant heterogeneity was observed in the analysis of MUHOW and MUHO individuals. This heterogeneity might be caused by differences in study characteristics, such as sample sizes, length of follow-up, genetic background, the equation used for estimating GFR, and the varied number of confounders controlled for in the studies. In addition to the mentioned reasons, the studies also differed in definitions used for metabolic health status and BMI categories, which may partly result in between-study heterogeneity when data were pooled. However, with the small number of studies currently available, subgroup analyses were impossible to be performed to find the source of heterogeneity. Finally, all included studies except two originated from Asian populations and data concerning other ethnicities such as Caucasians and Africans were not found. Therefore, further studies are warranted to examine the possible differences caused by ethnicity.

In conclusion, this meta-analysis of prospective studies supports the concept of heterogeneity of metabolic status among individuals within a similar BMI category. Our analysis indicated that MHOW and MHO phenotypes are positively in relation to CKD risk. The term ‘healthy’ might not be appropriate to describe these individuals because of the likelihood of underestimating the long-term effects of obesity, as well as less attention being paid to weight management. In addition, metabolically unhealthy phenotypes, including unhealthy normal weight, and unhealthy obese individuals had increased risk for CKD events, compared with MHNW individuals. Therefore, it is essential to consider both BMI and metabolic factors to reliably estimate the risk of incident CKD.
